# Supplementary material for: Molecular Epidemiology and Presence of Hybrid Pathogenic Escherichia coli among Isolates from Community-Acquired Urinary Tract Infection
Source: Microorganisms. 2022 Jan 27;10(2):302. doi: 10.3390/microorganisms10020302 (PMC8874565; doi:10.3390/microorganisms10020302)
Supplement: Supplementary file 1 [file microorganisms-10-00302-s001.zip › microorganisms-1535739-supplementary.pdf]

Supplemental material
Molecular epidemiology and presence of hybrid pathogenic *Escherichia coli* among isolates from community-acquired urinary tract infection

Júllia A. S. Nascimento[1], Fernanda F. Santos [2], José F. Santos-Neto[1], Liana O. Trovão[1], Tiago B. Valiatti[2], Isabel C. Pinaffi [3], Mônica A. M. Vieira[1], Rosa M. Silva[4], Ivan N. Falsetti [3], Ana C. M. Santos[1], Tânia A. T. Gomes [1]*

Table S1 Primers and conditions used for phylogenetic classification [a].

Reaction	Primers designation	Primers (5′ – 3′)	Cycles	Amplicon (bp)
Multiplex	chuA.1b<br>chuA.2	ATGGTACCGGACGAACCAAC<br>TGCCGCCAGTACCAAAGACA	94 °C – 4 min; 30 × (94 °C – 5 s and 59 °C – 20 s); 72 °C – 5 min	288
	yjaA.1b<br>yjaA.2b	CAAACGTGAAGTGTCAGGAG<br>AATGCGTTCCTCAACCTGTG		211
	TspE4C2.1b<br>TspE4C2.2b	CACTATTCGTAAGGTCATCC<br>AGTTTATCGCTGCGGGTCGC		152
	AceK.f<br>ArpA1.r	AACGCTATTCGCCAGCTTGC<br>TCTCCCCATACCGTACGCTA		400
phylogroup E	ArpAgpE.f<br>ArpAgpE.r	GATTCCATCTTGTCAAAATATGCC<br>GAAAAGAAAAAGAATTCCCAAGAG	94 °C – 4 min; 30 × (94 °C – 5 s and 57 °C – 20 s); 72 °C – 5 min	301
phylogroup C	trpAgpC.1<br>trpAgpC.2	AGTTTTATGCCCAGTGCGAG<br>TCTGCGCCGGTCACGCCC	94 °C – 4 min; 30 × (94 °C – 5 s and 59 °C – 20 s); 72 °C – 5 min	219
Control [b]	trpBA.f<br>trpBA.r	CGGCGATAAAGACATCTTCAC<br>GCAACGCGGCCTGGCGGAAG		489

[a] according to the method described by Clermont et al 2013 [33]. [b] used as internal PCR control in reaction for phylogroups C and E

Reference

33. Clermont, O.; Christenson, J.K.; Denamur, E.; Gordon, D.M. The Clermont *Escherichia coli* Phylo-Typing Method Revisited: Improvement of Specificity and Detection of New Phylo-Groups. *Environ. Microbiol. Rep.* **2013**, *5*, 58–65, doi:10.1111/1758-2229.12019.

Table S2. Primers and conditions used in PCR for amplification of *E. coli* virulence genes

Target gene	Primers (5′ – 3′)	Cycle	Product size (bp)	Reference
***afaBC*III**	F: GCTGGGCAGCAAACTGATAACTCTC<br>R: CATCAAGCTGTTTGTTCGTCCGCCG	94 °C – 5 min; 25 × (94 °C – 2 min; 65 °C – 1 min; 72 °C – 2 min); 72 °C – 8 min	750	[94]
***afaE*VIII**	F: CTAACTTGCCATGCTGTGACAGTA<br>R: TTATCCCCTGCGTAGTTGTGAATC	94 °C – 5 min; 25 × (94 °C – 2 min; 65 °C – 1 min; 72 °C – 2 min); 72 °C – 8 min	302	[95]
aggR	F: CGATACATTAAGACGCCTAAAG<br>R: TCTGATACATTAAATTCATCTGC	94 °C – 5 min; 30 × (94 °C – 1 min; 56 °C – 1 min; 72 °C – 1 min); 72 °C – 7 min	346	[96]
bfpB	F: GACACCTCATTGCTGAAGTCG<br>R: CCAGAACACCTCCGTTATGC	94 °C – 5 min; 30 × (94 °C – 30 s; 63 °C –30 s; 72 °C – 90 s); 72 °C – 5 min	910	[96]
bmaE	F: ATGGCGCTAACTTGCCATGCTG<br>R: AGGGGGACATATAGCCCCCTTC	95 °C – 12 min; 25 × (94 °C – 30 s; 63 °C – 30 s; 68 °C – 3 min); 72 °C – 10 min	507	[97]
cdtA	F: TTACTGGTTGCTCGAATGGGGATCGGT<br>R: GACAGGTGGACTTATTGCCCATAGC	94 °C – 5 min; 30 × (94 °C – 30 s; 55 °C – 30 s; 72 °C – 1 min); 72 °C – 5 min	575	[98]
cf29A	F: TCAACAACACTATCAAGGAA<br>R: ACCAAGTGCGTAAGAGGA	94 °C – 1 min; 30 × (94 °C – 30 s; 48 °C – 1 min; 72 °C – 90 s); 72 °C – 7 min	572	[99]
chuA	F: ATGGTACCGGACGAACCAAC<br>R: TGCCGCCAGTACCAAAGACA	94 °C – 4 min; 30 × (94 °C – 5 s; 59 °C – 20 s); 72°C – 5 min	288	[33]
cvaC	F: CACACACAAACGGGAGCTGTT<br>R: CTTCCCGCAGCATAGTTCCAT	95 °C – 12 min; 25 × (94 °C – 30 s; 63 °C – 30 s; 68 °C – 3 min); 72 °C – 10 min	680	[97]
daaE	F: GAACGTTGGTTAATGTGGGGTAA<br>R: TATTCACCGGTCGGTTATCAGT	94 °C – 5 min; 35 × (94°C – 90 s; 60°C – 90 s; 72°C – 90 s); 72°C – 5min	542	[100]
eae	F: CCCGGCACAAGCATAAGCTAA<br>R: ATGACTCATGOCCAGCTCA	95 °C – 5 min; 35 × (94 °C – 1 min; 55 °C – 2 min; 72°C – 1 min); 72 °C – 7 min	924	[101]
elt	F: GAACAGGAGGTTTCTGCGTTAGGTG<br>R: CTTTCAATGGCTTTTTTTTGGGAGTC	94 °C – 5 min; 30 × (94 °C – 30 s; 63 °C – 30 s; 72°C – 90 s); 72 °C – 5 min	655	[96]
escV	F: ATTCTGGCTCTCTTCTTCTTTATGGCTG<br>R: CGTCCCCTTTTACAAACTTCATCGC	94 °C – 5 min; 30 × (94 °C – 30 s; 63 °C – 30 s; 72 °C – 90 s); 72 °C – 5 min	544	[96]
estIa	F: CCTCTTTTAGYCAGACARCTGAATCASTTG<br>R: CAGGCAGGATTACAACAAAGTTCACAG	94 °C – 5 min; 30 × (94 °C – 30 s; 63 °C – 30 s; 72 °C – 90 s); 72 °C – 5 min	157	[96]
estIb	F:TGTCTTTTTCACCTTTCGCTC<br>R: CGGTACAAGCAGGATTACAACAC	94 °C – 5 min; 30 × (94 °C – 30 s; 63 °C – 30 s; 72 °C – 90 s); 72 °C – 5 min	171	[96]
fimA	F: GTTGATCAAACCGTTCAG<br>R: AATAACGCGCCTGGAACG	94 °C – 5 min; 27 × (94 °C – 45 s; 50 °C – 45 s; 72 °C – 1 min); 72 °C – 5 min	331	[102]
fyuA	F: GTAAACAATCTTCCCGCTCGGCAT<br>R: TGACGATTAACGAACCGGAAGGGA	95 °C –15 min; 30 × (94 °C – 30 s; 63 °C – 90 s; 72 ° C– 90 s); 72 °C – 10 min	850	[35]

hlyA	F: AACAAGGATAAGCACTGTTCTGGCT<br>R: ACCATATAAGCGGTCATTCCCGTCA	94 °C – 5 min; 30 × (94 °C – 1 min; 63 °C – 30 s; 72 °C – 3 min); 72 °C – 10 min	1177	[103]
hra	F: TCACTTGCAGACCAGCGTTTC<br>R: GTAACTCACACTGCTGTCACCT	94 °C – 3 min; 25 × (94 °C – 30 s; 58 °C – 30 s; 68 °C – 3 min); 72 °C – 10 min	537	[104]
iha	F: CAGGTCGGGGTTACCAAGT<br>R: CAAATGGCTCTCTTCCGTCAATGC	94 °C – 5 min; 30 × (94 °C – 1 min; 59 °C – 1 min; 72 °C – 1 min); 72 °C – 7 min	925	[105]
invE	F: CGATAGATGGCGAGAAATTATATCCCG<br>R: CGATCAAGAATCCCTAACAGAAGAATCAC	94 °C – 5 min; 30 × (94 °C – 30 s; 63 °C – 30 s; 72 °C – 90 s); 72 °C – 5 min	766	[96]
ireA	F: ATTGCCGTGATGTGTTCTGC<br>R: CACGGATCACTTCAATGCGT	94 °C – 3 min; 25 × (94 °C – 30 s; 58 °C – 30 s; 68 °C – 3 min); 72 °C – 10 min	384	[104]
iroN	F: AAGTCAAAGCAGGGGTTGCCCG<br>R: GACGCCGACATTAAGACGCAG	95 °C – 12 min; 25 × (94 °C – 30 s; 63 °C – 30 s; 68 °C – 3 min); 72 °C – 10 min	665	[106]
***kpsMT*III**	F: TCCTCTTGCTACTATTCCCCCT<br>R: AGGCGTATCCATCCCTCCTAAC	95 °C – 12 min; 25 × (94 °C – 30 s; 63 °C – 30 s; 68 °C – 3 min); 72 °C – 10 min	392	[97]
***kpsMT*II**	F: GCGCATTTGCTGATACTGTTG<br>R: CATCCAGACGATAAGCATGAGCA	95 °C – 12 min; 25 × (94 °C – 30 s; 63 °C – 30 s; 68 °C – 3 min); 72 °C – 10 min	272	[97]
mat	F: TATACGCTGGACTGAGTCGTG<br>R: CAGGTAGCGTCGAACTGTA	94 °C – 3 min; 25 × (94 °C – 30 s; 58 °C – 30 s; 68 °C – 3 min); 72 °C – 10 min	899	[104]
ompA	F: AGCTATCGCGATTGCAGTG<br>R: GGTGTTGCCAGTAACCGG	94 °C – 3 min; 25 × (94 °C – 30 s; 58 °C – 30 s; 68 °C – 3 min); 72 °C – 10 min	919	[104]
ompT	F: ATCTAGCCGAAGAAGGAGGC<br>R: CCCGGGTCATAGTGTTCATC	95 °C – 12 min; 25 × (94 °C – 30 s; 63 °C – 30 s; 68 °C – 3 min); 72 °C – 10 min	559	[107]
papC	F: GACGGCTGTACTGCAGGGTGTGGCG<br>R: ATATCCTTTCTGCAGGGATGCAATA	94°C – 5 min; 25 × (94°C – 2 min; 65°C – 1 min; 72°C – 2 min); 72°C – 8 min	328	[94]
pet	F: GTGTTTCAACCAGGTTCAACA<br>R: CCTTCACCAATTTTATGCAGT	95 °C – 15 min; 30 × (94 °C – 1 min; 52 °C – 1 min; 72°C – 90 s); 72 °C – 10 min	1037	[108]
pic	F: AGCCGTTTCCGCAGAAGCC<br>R: AAATGTCAGTGAACCGACGATTGG	94 °C – 5 min 30 × (94 °C – 30 s; 63°C – 30 s; 72 °C – 1,5 mim); 72°C – 5 min	1111	[96]
saa	F: CGTGATGAACAGGCTATTGC<br>R: ATGGACATGCCTGTGGCAAC	94 °C – 5 min; 30 × (94 °C – 30 s; 55 °C – 30 s; 72 °C – 30 s); 72°C – 5 min	119	[109]
sat	F: TGCTGGCTCTGGAGGAAC<br>R: TTGAACATTCAGAGTACCGGG	94 °C – 3 min; 25 × (94 °C – 30 s; 58 °C – 30 s; 68 °C – 3 min) 72 °C – 10 min	667	[104]
sfaDE	F: CGGAGGAGTAATTACAAACCTGGCA<br>R: CTCCGGAGAACTGGGTGCATCTTAC	94°C – 5 min; 25 × (94°C – 2 min; 65°C – 1 min; 72°C – 2 min); 72°C – 8 min	410	[94]
shf	F: ACTTTCTCCCGAGACATTC<br>R: CTTTAGCGGGAGCATTCAT	94 °C – 3 min; 25 × (94 °C – 30 s; 58 °C – 30 s; 68 °C – 3 min); 72 °C – 10 min	613	[110]
sitA	F: AGGGGGCACAACTGATTCTCG<br>R: TACCGGGCCGTTTTCTGTGC	94 °C – 5 min; 30 × (94 °C – 30 s; 59 °C – 30 s; 72 °C – 3 min); 72 °C – 10 min	608	[111]

stx1	F: CGATGTTACGGTTTGTTACTGTGACAGC<br>R: AATGCCACGCTTCCCAGAATTG	94 °C – 5 min; 30 × (94 °C – 30 s; 63 °C – 30 s; 72 °C – 90 s);<br>72 °C – 5min	244	[96]
stx2	F: GTTTTGACCATCTTCGTCTGATTATTGAG<br>R: AGCGTAAGGCTTCTGCTGTGAC	94 °C – 5 min; 30 × (94 °C – 30 s; 63 °C – 30 s; 72 °C – 90 s);<br>72 °C – 5 min	324	[96]
traT	F: GGTGTGGTGCGATGAGCACAG<br>R: CACGGTTCAGCCATCCCTGAG	95 °C – 12 min; 25 × (94 °C – 30 s; 63 °C – 30 s; 68 °C – 3<br>min); 72 °C – 10 min	290	[97]
tsh	F: GGTGGTGCACTGGAGTGG<br>R: AGTCCAGCGTGATAGTGG	94 °C – 3 min; 25 × (94 °C – 30 s; 58 °C – 30 s; 68 °C – 3 min);<br>72 °C – 10 min	620	[112]
vat	F: TCAGGACACGTTCAGGCATTCAGT<br>R: GGCCAGAACATTTGCTCCCTTGTT	95 °C –15 min; 30 × (94 °C – 30 s; 63 °C – 90 s; 72 °C– 90 s);<br>72 °C – 10 min	1100	[35]
yfcV	F: ACATGGAGACCACGTTCACC<br>R: GTAATCTGGAATGTGGTCAGG	95 °C –15 min; 30 × (94 °C – 30 s; 63 °C – 90 s; 72 °C– 90 s);<br>72 °C – 10 min	292	[35]

References

33. Clermont, O.; Christenson, J.K.; Denamur, E.; Gordon, D.M. The Clermont *Escherichia coli* Phylo-Typing Method Revisited: Improvement of Specificity and Detection of New Phylo-Groups. *Environ. Microbiol. Rep.* **2013**, *5*, 58–65. https://doi.org/10.1111/1758-2229.12019.
35. Spurbeck, R.R.; Dinh, P.C.; Walk, S.T.; Stapleton, A.E.; Hooton, T.M.; Nolan, L.K.; Kim, K.S.; Johnson, J.R.; Mobley, H.L.T. *Escherichia coli* Isolates That Carry *vat*, *fyuA*, *chuA*, and *yfcV* Efficiently Colonize the Urinary Tract. *Infect. Immun.* **2012**, *80*, 4115–4122. https://doi.org/10.1128/IAI.00752-12.
94. Le Bouguenec, C.; Archambaud, M.; Labigne, A. Rapid and specific detection of the *pap, afa, and sfa* adhesin-encoding operons in uropathogenic *Escherichia coli* strains by polymerase chain reaction. *J. Clin. Microbiol.* **1992**, 5, 1189–1193. doi: 10.1128/jcm.30.5.1189-1193.1992.
95. Lalioui, L.; Le Bouguénec, C. afa-8 Gene cluster is carried by a pathogenicity island inserted into the tRNA(Phe) of human and bovine pathogenic *Escherichia coli* isolates. *Infect. Immun.* **2001**, *69*, 937–948. doi: 10.1128/IAI.69.2.937-948.2001.
96. Müller, D.; Greune, L.; Heusipp, G.; Karch, H.; Fruth, A.; Tschäpe, H.; Schmidt, M.A. Identification of unconventional intestinal pathogenic *Escherichia coli* isolates expressing intermediate virulence factor profiles by using a novel single-step multiplex PCR. *Appl. Environ. Microbiol.* **2007**, *73*, 3380–3390. doi: 10.1128/AEM.02855-06.
97. Johnson, J.R.; Stell, A.L. Extended virulence genotypes of *Escherichia coli* strains from patients with urosepsis in relation to phylogeny and host compromise. *J. Infect. Dis.* **2000**, *181*, 261–272. doi: 10.1086/315217.
98. Okuda, J.; Kurazono, H.; Takeda, Y. Distribution of the cytolethal distending toxin A gene (*cdtA*) among species of *Shigella* and *Vibrio*, and cloning and sequencing of the *cdt* gene from *Shigella dysenteriae*. *Microb. Pathog.* **1995**, *18*, 167–172. doi: 10.1016/s0882-4010(95)90022-5.
99. Santos, A.C.; Zidko, A.C.; Pignatari, A.C.; Silva, R.M. Assessing the diversity of the virulence potential of *Escherichia coli* isolated from bacteremia in São Paulo, Brazil. *Braz. J. Med. Biol. Res.* **2013**, *46*, 968–973. doi: 10.1590/1414-431X20133184. Epub 2013 Oct 15.
100. Vidal, M.; Kruger, E.; Durán, C.; Lagos, R.; Levine, M.; Prado, V.; Toro, C.; Vidal R. Single multiplex PCR assay to identify simultaneously the six categories of diarrheagenic *Escherichia coli* associated with enteric infections. *J. Clin. Microbiol.* **2005**, *43*, 5362–5365. doi: 10.1128/JCM.43.10.5362-5365.2005.
101. Gannon, V.P.; Rashed, M.; King, R.K.; Thomas, E.J. Detection and characterization of the *eae* gene of Shiga-like toxin-producing *Escherichia coli* using polymerase chain reaction. *J. Clin. Microbiol.* **1993**, *31*, 1268–1274. doi: 10.1128/jcm.31.5.1268-1274.1993.
102. Marc, D.; Dho-Moulin, M. Analysis of the *fim* cluster of an avian O2 strain of *Escherichia coli*: serogroup-specific sites within *fimA* and nucleotide sequence of *fimI*. *J. Med. Microbiol.* **1996**, *44*, 444–452. doi: 10.1099/00222615-44-6-444.
103. Yamamoto, S.; Terai, A.; Yuri, K.; Kurazono, H.; Takeda, Y.; Yoshida, O. Detection of urovirulence factors in *Escherichia coli* by multiplex polymerase chain reaction. *FEMS Immunol. Med. Microbiol.* **1995**, *12*, 85–90. doi: 10.1111/j.1574-695X.1995.tb00179.x.
104. Ewers, C.; Li, G.; Wilking, H.; Kiessling, S.; Alt, K.; Antáo, E.M.; Laturnus, C.; Diehl, I.; Glodde, S.; Homeier, T., et al. Avian pathogenic, uropathogenic, and newborn meningitis-causing *Escherichia coli*: how closely related are they? *Int. J. Med. Microbiol.* **2007,** *297*, 163–176. doi: 10.1016/j.ijmm.2007.01.003.

105. Szalo, I.M.; Goffaux, F.; Pirson, V.; Piérard, D.; Ball, H.; Mainil, J. Presence in bovine enteropathogenic (EPEC) and enterohaemorrhagic (EHEC) *Escherichia coli* of genes encoding for putative adhesins of human EHEC strains. *Res. Microbiol.* **2002**, *153*, 653–658. doi: 10.1016/s0923-2508(02)01379-7.
106. Johnson, J.R.; Russo, T.A.; Tarr, P.I.; Carlino, U.; Bilge, S.S.; Vary, J.C.Jr.; Stell, A.L. Molecular epidemiological and phylogenetic associations of two novel putative virulence genes, *iha* and i*roN* (*E. coli*), among *Escherichia coli* isolates from patients with urosepsis. *Infect. Immun.* **2000**, *68*, 3040–3047. doi: 10.1128/IAI.68.5.3040-3047.2000.
107. Johnson, J.R.; O'Bryan, T.T.; Low, D.A.; Ling, G.; Delavari, P.; Fasching, C.; Russo, T.A.; Carlino, U.; Stell, A.L. Evidence of commonality between canine and human extraintestinal pathogenic *Escherichia coli* strains that express *papG* allele III. *Infect. Immun.* **2000**, *68*, 3327–3336. doi: 10.1128/IAI.68.6.3327-3336.2000.
108. Gioppo, N.M.; Elias, W.P.Jr; Vidotto, M.C.; Linhares, R.E.; Saridakis, H.O.; Gomes, T.A.; Trabulsi, L.R.; Pelayo, J.S. Prevalence of HEp-2 cell-adherent *Escherichia coli* and characterisation of enteroaggregative *E. coli* and chain-like adherent *E. coli* isolated from children with and without diarrhoea, in Londrina, Brazil. *FEMS Microbiol. Lett.* **2000**, *190*, 293–298. doi: 10.1111/j.1574-6968.2000.tb09301.x.
109. Paton, A.W.; Paton, J.C. Direct detection and characterization of Shiga toxigenic *Escherichia coli* by multiplex PCR for *stx1, stx2, eae, ehxA,* and *saa. J. Clin. Microbiol.* **2002**, *40*, 271–274. doi:10.1128/JCM.40.1.271-274.2002
110. Czeczulin, J.R.; Whittam, T.S.; Henderson, I.R.; Navarro-Garcia, F.; Nataro, J.P. Phylogenetic analysis of enteroaggregative and diffusely adherent *Escherichia coli*. *Infect. Immun.* **1999** 67(6):2692-9. doi: 10.1128/IAI.67.6.2692-2699.1999.
111. Rodriguez-Siek, K.E.; Giddings, C.W.; Doetkott, C.; Johnson, T.J.; Nolan, L.K. Characterizing the APEC pathotype. *Vet. Res.* **2005**, *36*, 241–256. doi: 10.1051/vetres:2004057.
112. Delicato, E.R.; de Brito, B.G.; Konopatzki, A.P.; Gaziri, L.C.; Vidotto, M.C. Occurrence of the temperature-sensitive hemagglutinin among avian *Escherichia coli*. *Avian Dis.* **2002**, *46*, 713–716. doi: 10.1637/0005-2086(2002)046[0713:OOTTSH]2.0.CO;2.

Table S3. Virulence profile, phylogenetic origin, and characterization of intrinsic virulence and uropathogenic potential of strains isolated from urine in the Mogi Guaçu community

**Strains** [a]	Virulence profile[b]	Phylogroup [c]	ExPEC+ [d]	UPEC+ [d]
**LSC 001**	*fimA, ompT, ompA, hra, sitA, mat, kpsMT*III, *saa, cdtA, iroN,* ***papC, kpsMT*II*, vat, fyuA, yfcV, chuA***	B2	+	+
**LSC 002**	*fimA, ompT, ompA, hra, sitA, sat, iha, mat, ireA, traT,* ***iucD, fyuA, yfcV, chuA***	B2	-	-
**LSC 003**	*fimA, ompT, ompA, hra, sitA, kpsMTIII, mat, ireA, iroN, traT,* ***papC, vat, fyuA, yfcV, chuA***	B2	-	+
**LSC 004**	*fimA, ompT, ompA, hra, sitA, sat, iha, mat, ireA, saa, iroN,* ***papC, kpsMT*II*, iucD, vat, fyuA, yfcV, chuA***	B2	+	+
**LSC 005**	*fimA, ompA, ompT, sitA, mat, traT,* ***chuA***	E	-	-
**LSC 006**	*fimA, ompA, ompT, mat*	A	-	-
**LSC 007**	*fimA, ompA, shf, ireA, traT,* ***fyuA***	A	-	-
**LSC 008**	*fimA, ompA, ompT, mat*	A	-	-
**LSC 009**	*fimA, ompA, hra, sitA, mat, tsh, ireA, saa, iroN, traT,* ***iucD, vat, fyuA, yfcV, chuA***	B2	-	+
**LSC 010**	*fimA, ompA, ompT, cvaC, sitA, mat, saa, pet,* ***kpsMT*II*, vat, fyuA, yfcV, chuA***	NC [e]	-	+
**LSC 011**	*fimA, ompA, hra, ompT, sitA, sat, iha, mat, pet, traT,* ***iucD, fyuA, yfcV, chuA***	B2	-	-
**LSC 012**	*fimA, ompA, sitA, sat, iha, saa, pet, daaE, traT,* ***sfaDE, iucD, fyuA, yfcV, chuA***	B2	+	-
**LSC 013**	*fimA, ompA, ompT, sitA, sat, iha, mat, ireA, pet, traT,* ***iucD, fyuA, yfcV, chuA***	B2	-	-
**LSC 014**	*fimA, ompA, hra, ompT, sitA, sat, iha, pic, ireA, saa, pet, iroN,* ***papC, iucD, chuA***	B2	+	-
**LSC 015**	*fimA, ompA, traT,* ***kpsMT*II**	A	-	-
**LSC 016**	*fimA, ompA, sitA, traT*	A	-	-
**LSC 017**	*fimA, ompA, hra, ompT, sitA, saa, iroN, traT,* ***papC, chuA***	B2	-	-
**LSC 018**	*fimA, ompA, ompT, sitA, pic,* ***chuA***	B2	-	-
**LSC 019**	*fimA, mat*	A	-	-
**LSC 020**	*fimA, ompT, sitA, pic, iroN, traT,* ***papC, kpsMT*II*, vat, fyuA, yfcV, chuA***	B2	+	+
**LSC 021**	*fimA, sitA, traT*	NC	-	-
**LSC 022**	*fimA, sitA,* ***iucD, fyuA***	A	-	-
**LSC 023**	*fimA, ompT, sitA, iroN, traT,* ***iucD, vat, fyuA, chuA***	NC	-	-
**LSC 024**	*fimA*	C	-	-
**LSC 025**	*fimA,* ***kpsMT*II*, chuA***	D	-	-
**LSC 026**	*fimA, ompT, sitA, sat, iha, ireA, iroN, traT,* ***kpsMT*II*, vat, fyuA, yfcV, chuA***	B2	-	+
**LSC 027**	*fimA, sitA, shf*	C	-	-

Table S3. Virulence profile, phylogenetic origin, and characterization of intrinsic virulence and uropathogenic potential of strains isolated from urine in the Mogi Guaçu community. Continued

Strains [a]	Virulence profile[b]	Phylogroup [c]	ExPEC+ [d]	UPEC+ [d]
**LSC 028**	*fimA, ompT, ompA, sitA, pic, shf, saa, traT,* ***vat, fyuA, yfcV, chuA***	B2	-	+
**LSC 029**	*fimA, ompT, ompA, iroN, traT,* ***iucD***	B1	-	-
**LSC 030**	*fimA, ompA, hra, sitA, iroN, traT*	B1	-	-
**LSC 031**	*fimA, ompT, ompA, saa, pet, traT,* ***vat, fyuA, yfcV, chuA***	B2	-	+
**LSC 032**	*fimA, ompT, ompA, hra, sitA, sat, iha, traT,* ***chuA***	E	-	-
**LSC 033**	*fimA, ompT, ompA, hra, sitA, saa, traT,* ***papC, kpsMTII, sfaDE, iucD, vat, fyuA, yfcV, chuA***	B2	+	+
**LSC 034**	*fimA, ompT, ompA, pet, iroN,* ***kpsMTII, vat, fyuA, yfcV, chuA***	B2	-	+
**LSC 036**	*fimA, saa, sitA, ompT, iroN, traT,* ***papC, sfaDE, kpsMTII, vat, fyuA, yfcV, chuA***	B2	+	+
**LSC 037**	*fimA, ompA,* ***chuA***	B2	-	-
**LSC 038**	*fimA, sitA, ompT,* ***papC, kpsMTII, fyuA, chuA***	D	+	-
**LSC 039**	*fimA, shf, ompA, traT,* ***papC, iucD***	B1	+	-
**LSC 040**	*fimA, sitA, cvaC, ompA, iroN, traT,* ***fyuA***	B1	-	-
**LSC 041**	*fimA, ompT, ompA,* ***sfaDE, chuA***	F	-	-
**LSC 042**	*fimA, sitA, cvaC, ompA, iroN, traT*	C	-	-
**LSC 043**	*fimA, sitA, ompT, ompA, iroN,* ***papC, sfaDE, kpsMTII, vat, fyuA, yfcV, chuA***	B2	+	+
**LSC 044**	*fimA, shf, sitA, sat, iha, ompT, ompA,* ***kpsMTII, vat, fyuA, yfcV, chuA***	B2	-	+
**LSC 045**	*fimA, saa, cdtA, sitA, sat, iha, ompT, ompA, hra, traT,* ***papC, iucD, kpsMTII, vat, fyuA, yfcV, chuA***	B2	+	+
**LSC 046**	*fimA, ompA, traT,* ***papC, fyuA***	A	-	-
**LSC 047**	*fimA, sitA, ompA, hra, iroN, traT, ompT,* ***sfaDE, kpsMTII, vat, fyuA, yfcV, chuA***	B2	+	+
**LSC 048**	*fimA, ompA*	A	-	-
**LSC 049**	*fimA,saa, sitA, cvaC, ompT, ompA, iroN, traT,* ***papC, kpsMTII, vat, fyuA, yfcV, chuA***	B2	+	+
**LSC 050**	*fimA, sitA, iroN, traT, ompT,* ***papC, sfaDE, kpsMTII, vat, fyuA, yfcV, chuA***	B2	+	+
**LSC 051**	*fimA, shf, sitA, traT*	A	-	-
**LSC 052**	*fimA, ompA, sitA, traT,* ***aggR, kpsMTII, chuA***	D	-	-
**LSC 053**	*fimA, ompA, hra, sitA, iroN, tratT, ompT, cvaC,* ***iucD, kpsMTII, vat, fyuA, yfcV, chuA***	B2	+	+
**LSC 054**	*fimA, ompA, hra, sitA, saa, iroN, tratT, ompT,* ***sfaDE, kpsMTII, vat, fyuA, yfcV, chuA***	B2	+	+
**LSC 055**	*fimA, ompA, hra, sitA, iroN, tratT,* ***iucD***	A	-	-

Table S3. Virulence profile, phylogenetic origin, and characterization of intrinsic virulence and uropathogenic potential of strains isolated from urine in the Mogi Guaçu community. Continued

**Strains** [a]	Virulence profile[b]	Phylogroup [c]	ExPEC+ [d]	UPEC+ [d]
**LSC 056**	*fimA, ompA, hra, sitA, sat, traT, ompT,* ***papC, iucD, kpsMTII, fyuA, yfcV, chuA***	B2	+	-
**LSC 057**	*fimA, ompA, hra, sitA, sat, traT, ompT,* ***papC, iucD, kpsMTII, fyuA, yfcV, chuA***	B2	+	-
**LSC 058**	*fimA, ompA, sitA, sat, hlyA, ompT,* ***iucD, kpsMTII, fyuA, yfcV, chuA***	B2	+	-
**LSC 059**	*fimA, ompA, hra, sitA, iroN, tratT, cvaC,* ***papC, iucD, kpsMTII, vat, fyuA, yfcV, chuA***	B2	+	+
**LSC 060**	*fimA, ompA, hra, ompT,* ***vat, fyuA, yfcV, chuA***	B2	-	+
**LSC 061**	*fimA, ompA, traT*	A	-	-
**LSC 062**	*fimA, ompA, hra, hlyA, ompT,* ***sfaDE, kpsMTII, chuA***	B2	+	-
**LSC 063**	*fimA, ompA, sitA, ompT,* ***papC, iucD***	C	+	-
**LSC 064**	*fimA, ompA, hra,*	A	-	-
**LSC 065**	*fimA, ompA, sitA, sat, traT, ompT,* ***papC, iucD, kpsMTII, chuA***	B2	+	-
**LSC 066**	*fimA, ompA, hra, traT*	A	-	-
**LSC 067**	*fimA, ompA,sitA, sat, saa, ompT,* ***papC, kpsMTII, chuA***	B2	+	-
**LSC 068**	*fimA, ompA, saa, ompT,* ***chuA***	F	-	-
**LSC 069**	*fimA, ompA, sitA, sat, iroN, tratT, hlyA, ompT,* ***sfaDE, kpsMTII, chuA***	B2	+	-
**LSC 070**	*fimA, ompA, iroN, tratT, cvaC,* ***papC, iucD, kpsMTII, fyuA, yfcV, chuA***	B2	+	-
**LSC 071**	*fimA, ompA, ompT*	A	-	-
**LSC 072**	*fimA, ompA, hra,* ***chuA***	B2	-	-
**LSC 073**	*fimA, ompA, ompT,* ***eae, escV, vat, fyuA, yfcV, chuA***	B2	-	+
**LSC 074**	*fimA, ompA, iroN, tratT, ompT,* ***sfaDE***	B1	-	-
**LSC 075**	*fimA, ompA, iroN, tratT, ompT,* ***papC, sfaDE, kpsMTII, chuA***	B2	+	-
**LSC 076**	*fimA, ompA, hra, shf,* ***fyuA, yfcV***	B1	-	-
**LSC 077**	*fimA, ompA, traT, ompT,* ***chuA***	B2	-	-
**LSC 078**	*fimA, ompA, cdtA,* ***kpsMTII, fyuA, yfcV***	B1	-	-
**LSC 079**	*fimA, iroN, tratT, hlyA, ompT,* ***sfaDE, kpsMTII, fyuA, chuA***	B2	+	-
**LSC 080**	*fimA, mat, hlyA, ompT,* ***fyuA, yfcV***	C	-	-
**LSC 081**	*fimA, mat, daaE, traT, ompT,* ***papC, kpsMTII, chuA***	D	+	-
**LSC 082**	*fimA, mat, ompA, hra, ompT,* ***kpsMTII, fyuA, chuA***	F	-	-

Table S3. Virulence profile, phylogenetic origin, and characterization of intrinsic virulence and uropathogenic potential of strains isolated from urine in the Mogi Guaçu community. Continued

**Strains** [a]	Virulence profile[b]	Phylogroup [c]	ExPEC+ [d]	UPEC+ [d]
**LSC 083**	*fimA, saa, pet, ompA, hra, hlyA, traT, ompT,* ***papC, kpsMT*****II,** ***chuA***	B2	+	-
**LSC 084**	*fimA, mat, ompA, iroN, ompT,* ***fyuA, yfcV, chuA***	B2	-	-
**LSC 085**	*fimA, mat, traT, ompT,* ***papC, iucD, kpsMT*****II,** ***fyuA, yfcV, chuA***	E	+	-
**LSC 087**	*fimA, mat, ompA, hra, traT, ompT,* ***kpsMT*****II,** ***fyuA, chuA***	E	-	-
**LSC 088**	*fimA, mat, traT,* ***papC, fyuA***	A	-	-
**LSC 089**	*fimA, saa, mat, pet, cdtA, ompA, hra, hlyA, iroN, traT, ompT,* ***papC, kpsMT*****II,** ***fyuA, chuA***	B2	+	-
**LSC 090**	*fimA, mat, ompA, ompT,* ***vat, fyuA, yfcV***	C	-	-
**LSC 091**	*fimA, mat, ompA, hra,* ***papC, iucD***	C	+	-
**LSC 092**	*fimA, ompA, hra,* ***papC, fyuA***	C	-	-
**LSC 093**	*fimA, saa, iroN, hlyA, pet, ompA, ompT,* ***sfaDE, kpsMT*****II,** ***fyuA, chuA***	B2	+	-
**LSC 094**	*fimA, traT, shf, ompA,* ***vat, fyuA, yfcV***	B1	-	-
**LSC 096**	*fimA,* ***fyuA***	A	-	-
**LSC 097**	*fimA, saa, iroN, hlyA, ompA,* ***sfaDE, chuA***	B2	-	-
**LSC 098**	*tratT, ompA,* ***vat, fyuA, yfcV***	A	-	-
**LSC 099**	*fimA, saa, ompA, hra*	B1	-	-
**LSC 100**	*fimA, saa, iroN, tratT, hlyA, pet, mat ompA, hra, ompT,* ***papC, sfaDE, kpsMT*****II,** ***chuA***	B2	+	-
**LSC 101**	*fimA, saa, iroN, hlyA, pet, cdtA, mat ompA, hra, ompT,* ***papC, sfaDE, kpsMT*****II,** ***vat, fyuA, yfcV, chuA***	B2	+	+
**LSC 102**	*fimA, iroN, tratT, hlyA, pet, mat, ompA, hra,* ***papC, sfaDE, vat, fyuA, yfcV, chuA***	B2	+	+
**LSC 103**	*iroN, tratT, mat, ompA, ompT,* ***kpsMT*****II,** ***vat, fyuA, yfcV, chuA***	B2	-	+
**LSC 104**	*iroN, tratT, hlyA, mat, ompA, hra, ompT,* ***iucD, kpsMT*****II,** ***vat, fyuA, yfcV, chuA***	B2	+	+
**LSC 105**	*fimA, tratT, hlyA, pet, mat ompA, hra, ompT,* ***papC, kpsMT*****II,** ***vat, fyuA, yfcV, chuA***	B2	+	+
**LSC 106**	*fimA, saa, traT, mat, ompA, ompT,* ***iucD, kpsMT*****II,** ***fyuA, yfcV***	C	+	-
**LSC 107**	*fimA, saa, iroN, tratT, mat, ireA, ompA,* ***papC, sfaDE, chuA***	B2	+	-
**LSC 108**	*fimA, iroN, tratT, hlyA, pet, mat, ompA, hra, cvaC, ompT,* ***iucD, kpsMT*****II,** ***vat, fyuA, yfcV, chuA***	B2	+	+
**LSC 109**	*fimA, iroN, tratT, pet, mat, ompA, ompT,* ***kpsMT*****II,** ***vat, yfcV, chuA***	B2	-	-
**LSC 110**	*shf, mat, ompA, cvaC, ompT,* ***iucD, kpsMT*****II,** ***fyuA, yfcV***	B1	+	-
**LSC 111**	*fimA, iroN, tratT, shf, mat, ompA, hra, cvaC,* ***kpsMT*****II**	B1	-	-

Table S3. Virulence profile, phylogenetic origin, and characterization of intrinsic virulence and uropathogenic potential of strains isolated from urine in the Mogi Guaçu community. Continued

**Strains** [a]	Virulence profile[b]	Phylogroup [c]	ExPEC+ [d]	UPEC +[d]
**LSC 112**	*fimA, saa, pet, mat, ireA, ompA, hra, ompT,* ***papC, iucD, kpsMT*****II*****, fyuA, chuA***	B2	+	-
**LSC 113**	*fimA, mat,* ***vat, fyuA, yfcV***	NC	-	-
**LSC 114**	*fimA, iroN, tratT, ompT, mat, ireA,* ***papC, kpsMT*****II*****, chuA***	E	+	-
**LSC 115**	*fimA, iroN, pic, ompT, hra, mat, ireA,* ***sfaDE, kpsMT*****II*****, chuA***	B2	+	-
**LSC 116**	*fimA, ompT, sitA, mat,* ***iucD, kpsMT*****II*****, yfcV, chuA***	B2	+	-
**LSC 117**	*fimA, ompA, mat,* ***yfcV***	NC	-	-
**LSC 118**	*fimA, iroN, tratT, pic, ompT, ompA, hra, sitA, mat,* ***sfaDE, kpsMT*****II*****, chuA***	B2	+	-
**LSC 119**	*fimA, iroN, cvaC, ompT, ompA, hra, sitA, mat,* ***papC, sfaDE, iucD, kpsMT*****II*****, yfcV, chuA***	B2	+	-
**LSC 120**	*fimA, ompT, sitA, mat,* ***papC, iucD, yfcV***	C	+	-
**LSC 121**	*fimA, saa, iroN, traT, ompT, sitA, mat,* ***papC, sfaDE, kpsMT*****II*****, chuA***	B2	+	-
**LSC 122**	*fimA, sitA, mat,* ***yfcV***	A	-	-
**LSC 123**	*fimA, saa, traT, ompT, ompA, sitA, mat,* ***kpsMT*****II*****, chuA***	B2	-	-
**LSC 124**	*fimA, ompT, ompA, hra, sitA, sat, iha, mat,* ***papC, iucD, kpsMT*****II**, ***yfcV, chuA***	B2	+	-
**LSC 126**	***iucD, vat, fyuA, yfcV***	NC	-	-
**LSC 127**	*fimA, iroN, hlyA, pic, ompT, ompA, hra, sitA, mat,* ***iucD, kpsMT*****II*****, chuA***	B2	+	-
**LSC 128**	*fimA, saa, iroN, tratT, cvaC, ompT, ompA, sitA, mat, ireA,* ***papC, iucD, kpsMT*****II*****, fyuA, yfcV, chuA***	B2	+	-
**LSC 129**	*fimA, saa, iroN, hlyA, pic, ompT, hra, sitA, sat, iha, mat, ireA,* ***papC, sfaDE, iucD, kpsMT*****II*****, fyuA, yfcV, chuA***	B2	+	-
**LSC 131**	*fimA, ompA, mat,* ***papC***	A	-	-
**LSC 132**	*fimA, iroN, ompT, ompA, mat,* ***iucD, fyuA***	B1	-	-
**LSC 133**	*fimA, ompA*	B1	-	-
**LSC 134**	*fimA, ompA,* ***yfcV, chuA***	B2	-	-
**LSC 135**	*ompA, mat*	A	-	-
**LSC 136**	*fimA, saa, iroN, hlyA, pic, ompT, ompA, hra, sitA, mat,* ***sfaDE, kpsMT*****II*****, fyuA, yfcV, chuA***	B2	+	-
**LSC 137**	*fimA, saa, ompT, ompA, hra, sitA, mat,* ***papC, sfaDE, kpsMT*****II*****, fyuA, yfcV, chuA***	B2	+	-
**LSC 138**	*fimA, ompA, hra, sitA, iha, mat,* ***fyuA***	A	-	-
**LSC 139**	*ompA, mat*	A	-	-
**LSC 140**	*fimA, ompT, ompA, sitA,* ***chuA***	F	-	-

Table S3. Virulence profile, phylogenetic origin, and characterization of intrinsic virulence and uropathogenic potential of strains isolated from urine in the Mogi Guaçu community. Continued

**Strains** [a]	Virulence profile[b]	Phylogroup [c]	ExPEC+ [d]	UPEC + [d]
**LSC 141**	*fimA, ompT, ompA, sitA, mat,* ***kpsMT*****II,** ***chuA***	E	-	-
**LSC 142**	*traT, shf, ompA, sitA, mat,* ***iucD***	A	-	-
**LSC 143**	*fimA, ompA, mat*	B1	-	-
**LSC 144**	*fimA, ompT, ompA, sitA, mat,* ***kpsMT*****II,** ***fyuA, yfcV, chuA***	B2	-	-
**LSC 145**	*fimA, iroN, tratT, cvaC, ompT, ompA, sitA, mat,* ***papC, fyuA***	C	-	-
**LSC 146**	*fimA, saa, ompT, ompA, hra, sitA, mat,* ***kpsMT*****II,** ***fyuA, yfcV, chuA***	B2	-	-
**LSC 147**	*fimA, iroN, cvaC, ompT, ompA, sitA,* ***sfaDE***	NC	-	-
**LSC 148**	*fimA, shf, ompT, ompA, sitA, mat,* ***iucD, fyuA***	B1	-	-
**LSC 149**	*fimA, ompT, ompA, sitA, mat*	NC	-	-
**LSC 150**	*fimA, ompT, ompA, sitA, sat, iha, mat,* ***kpsMT*****II,** ***fyuA, yfcV, chuA***	B2	-	-
**LSC 152**	*fimA, ompT, ompA, mat,* ***kpsMT*****II,** ***fyuA***	NC	-	-
**LSC 153**	*fimA, sitA, sat, ompA*	NC	-	-
**LSC 154**	*fimA, ompA, hra*	NC	-	-
**LSC 155**	*fimA, ompT, ompA, hra, mat,* ***iucD, kpsMT*****II,** ***vat, fyuA, yfcV, chuA***	B2	+	+
**LSC 156**	*fimA, iroN, ompT, ompA, sitA, mat,* ***fyuA***	C	-	-
**LSC 158**	*fimA, iroN, ompT, ompA, sitA, cdtA, mat,* ***kpsMT*****II,** ***vat, fyuA, yfcV, chuA***	B2	-	+
**LSC 160**	*fimA, iroN, ompA, mat,* ***sfaDE, yfcV, chuA***	B2	-	-
**LSC 161**	*fimA, ompT, ompA, hra, sitA, mat,* ***kpsMT*****II,** ***fyuA, yfcV, chuA***	B2	-	-
**LSC 162**	*fimA, ompT, ompA, hra,* ***kpsMT*****II,** ***fyuA, chuA***	E	-	-
**LSC 163**	*fimA, ompT, ompA, hra,* ***fyuA, yfcV, chuA***	F	-	-
**LSC 164**	*ompA, hra*	C	-	-
**LSC 165**	*fimA, iroN, ompT, ompA, hra*	B1	-	-
**LSC 166**	*fimA, ompA, hra*	B1	-	-
**LSC 168**	*fimA, ompT, ompA, hra, sitA, iha,* ***papC, kpsMT*****II,** ***fyuA, yfcV, chuA***	B2	+	-
**LSC 169**	*fimA, saa, ompT, ompA, sitA, iha*	B1	-	-
**LSC 170**	*fimA, shf, saa, ompA, hra,* ***kpsMT*****II,** ***fyuA, chuA***	E	-	-
**LSC 171**	*fimA, saa, cvaC, ompT, ompA, sitA, sat, iha,* ***fyuA***	B1	-	-

Table S3. Virulence profile, phylogenetic origin, and characterization of intrinsic virulence and uropathogenic potential of strains isolated from urine in the Mogi Guaçu community. Continued

**Strains** [a]	Virulence profile[b]	Phylogroup [c]	ExPEC+ [d]	UPEC+ [d]
**LSC 172**	*fimA, shf, saa, ompT, ompA, sitA*	B1	-	-
**LSC 173**	*fimA, ompA, sitA,* ***fyuA***	A	-	-
**LSC 174**	*fimA, ireA, ompA, sitA,* ***fyuA, yfcV***	NC	-	-
**LSC 175**	*fimA, ompT, ompA, sitA,* ***kpsMTII, fyuA***	A	-	-
**LSC 176**	*fimA, ompA, sitA, sat, iha*	A	-	-
**LSC 178**	*fimA, ompT, ompA, sitA,* ***yfcV, chuA***	B2	-	-
**LSC 180**	*fimA, cvaC, ompT, ompA, sitA,* ***kpsMTII, fyuA, chuA***	E	-	-
**LSC 182**	*fimA, ompT, sitA, iha,* ***kpsMTII***	NC	-	-
**LSC 183**	*fimA, ompA, sitA,* ***eae, escV***	B1	-	-
**LSC 184**	*fimA, ompA, hra, sitA,* ***papC***	C	-	-

[a] The hybrid *E. coli* strains identified are shown in red; [b] VFs related to molecular classification into ExPEC+ (at least two among *papC, afaBCIII, sfaDE, iucD, kpsMT*II) , UPEC+ (simultaneous presence of *vat, yfcV, chuA, fyuA*) or DEC (*aggR, eae, escV*) are in bold; [c] According to the multiplex PCR described by Clermont et al. 2013; [d] ExPEC+ means intrinsic virulence; UPEC+ means uropathogenic potential; [e] NC – not classified; (+) strains that fulfill the assessed criterium; (-) strains that do not fulfill the assessed criterium.

Table S4. Frequency of phylogroups and molecular classification markers regarding occurrence of UTI symptoms [n(%)]

Traits	ABU (n=36)	UTI (n=94)
**Phylogroups**		
A	8 (22.2)	12 (12.7)
B1	5 (13.8)	12 (12.7)
B2	13 (36.1)	44 (46.8)
C	4 (11.1)	6 (6.3)
D	0 (0)	4 (4.2)
E	1 (2.7)	6 (6.3)
F	1 (2.7)	4 (4.2)
NC	4 (11.1)	5 (5.3)
**Molecular classification** [a]		
ExPEC+	12 (33.3)	33 (35.1)
UPEC+	7 (19.4)	16 (17)
DEC	2 (5.5)	1 (1)

[a] The molecular classification was performed as follows: intrinsic virulence (ExPEC+), presence of at least two of the genes *papC, afaBC*III, *sfaDE*, *iucD*, and *kpsMT*II; uropathogenic potential (UPEC+), the simultaneous presence of *vat, chuA, fyuA,* and *yfcV*; DEC, presence of a virulence marker used in the molecular diagnosis of the DEC pathotypes (*eae, escV, bfpB, aggR, stx1, stx2, eltA, estA,* and *invE*). ABU – Asymptomatic bacteriuria; UTI – Urinary tract infection. Groups were compared using Fisher Exact test ($P < 0.05$) and no difference was identified. NC, non-classifiable by Clermont method.
